# The miRNAs *let-7b* and *miR-141* Coordinately Regulate Vitellogenesis by Modulating Methyl Farnesoate Degradation in the Swimming Crab *Portunus trituberculatus*

**DOI:** 10.3390/ijms25010279

**Published:** 2023-12-24

**Authors:** Xuee Yu, Mengqian Zhang, Ping Liu, Jitao Li, Baoquan Gao, Xianliang Meng

**Affiliations:** 1 State Key Laboratory of Mariculture Biobreeding and Sustainable Goods, Yellow Sea Fisheries Research Institute, Chinese Academy of Fishery Sciences, Qingdao 266071, China; 2 Laboratory for Marine Fisheries Science and Food Production Processes, Laoshan Laboratory, Qingdao 266237, China; 3 Key Laboratory of Aquatic Genomics, Ministry of Agriculture and Rural Affairs, Yellow Sea Fisheries Research Institute, Chinese Academy of Fishery Sciences, Qingdao 266071, China

**Keywords:** miRNA, methyl farnesoate, vitellogenesis, ovary, crab

## Abstract

Methyl farnesoate (MF), a crucial sesquiterpenoid hormone, plays a pivotal role in the reproduction of female crustaceans, particularly in the vitellogenesis process. Despite extensive research on its functions, the molecular mechanisms that regulate MF levels during the vitellogenic phase remain largely elusive. This study investigates the roles of microRNAs (miRNAs), significant post-transcriptional regulators of gene expression, in controlling MF levels in the swimming crab *Portunus trituberculatus*. Through bioinformatic analysis, four miRNAs were identified as potential regulators targeting two genes encoding Carboxylesterases (CXEs), which are key enzymes in MF degradation. Dual luciferase reporter assays revealed that *let-7b* and *miR-141* suppress *CXE1* and *CXE2* expression by directly binding to their 3′ UTRs. In vivo overexpression of *let-7b* and *miR-141* significantly diminished *CXE1* and *CXE2* levels, consequently elevating hemolymph MF and enhancing *vitellogenin* expression. Spatiotemporal expression profile analysis showed that these two miRNAs and their targets exhibited generally opposite patterns during ovarian development. These findings demonstrate that *let-7b* and *miR-141* collaboratively modulate MF levels by targeting *CXEs*, thus influencing vitellogenesis in *P. trituberculatus*. Additionally, we found that the expression of *let-7b* and *miR-141* were suppressed by MF, constituting a regulatory loop for the regulation of MF levels. The findings contribute novel insights into miRNA-mediated ovarian development regulation in crustaceans and offer valuable information for developing innovative reproduction manipulation techniques for *P. trituberculatus*.

## 1. Introduction

Vitellogenesis is a critical event in female reproduction of oviparous animals [1]. In decapod crustaceans, vitellogenesis is controlled by various endocrine regulators including neuropeptides, ecdysteroids, and methyl farnesoate (MF). Of these, MF is particularly significant in controlling vitellogenesis [2]. MF is a sesquiterpenoid with a structural similarity to juvenile hormone (JH) III in insects, often considered its crustacean counterpart. Like JH III, there is accumulating evidence that MF serves as a potent gonadotropin in crustaceans [3,4]. For instance, the levels of MF in the hemolymph have been positively linked to vitellogenesis in various decapod species [5,6]. Moreover, increased MF levels, achieved through direct injection or dietary administration, have been shown to promote vitellogenin production and oocyte maturation [7,8,9]. Given its pivotal role in the reproductive development of female crustaceans, extensive research has sought to elucidate the regulatory mechanisms of MF levels. It is well established that the MF level is under the negative control of neuropeptides from the sinus gland/X-organ complex (SG/XO) in the eyestalk, termed mandibular organ inhibiting hormone (MOIH) [10]. The MOIH appears to regulate MF by inhibiting the enzyme Farnesoic acid O-methyltransferase (FAMeT), which is responsible for the final step in MF biosynthesis [6]. Recently, the Carboxylesterases (CXEs) responsible for degrading MF have been identified in several decapods [11,12,13]. The expression of these enzymes correlates with vitellogenesis and can be stimulated by MF treatment in vivo, suggesting their involvement in vitellogenesis through the modulation of MF degradation. Despite advances in understanding MF metabolism regulation, the complete molecular mechanisms that govern MF levels during vitellogenesis in crustaceans remain to be fully elucidated.

MicroRNAs (miRNAs) are a category of endogenous small non-coding RNAs that play a pivotal role in the regulation of gene expression at the post-transcriptional level [14]. In animals, miRNAs function by binding to the 3′ untranslated regions (UTRs) of target mRNAs, which leads to translational repression or mRNA degradation [15]. These molecules are crucial in controlling a variety of physiological processes, including female reproduction [16]. For example, in insects, specific miRNAs have been linked to the regulation of vitellogenesis. In the migratory locust *Locusta migratoria*, *miR-2*, *miR-13*, and *miR-71* collectively regulate the notch signaling pathway to modulate vitellogenin mRNA expression, while *let-7* and *miR-278* influence vitellogenesis by targeting the juvenile hormone early-response gene *Kruppel-homolog 1* (*Kr-h1*) [17,18]. In the silkworm *Bombyx mori*, *miR-2739* and a newly identified miRNA (novel-miR-167) are involved in regulating the *vitellogenin receptor*, which is essential for vitellogenin endocytosis in the ovary [19]. In the hard tick *Haemaphysalis longicornis*, *miR-275* has been shown to directly affect *vitellogenin* expression [20].

With the advancement of high-throughput sequencing technologies, the expression profiles of gonadal miRNAs have been extensively characterized in several decapods, such as *Scylla paramamosain* [21,22], *Portunus trituberculatus* [23], *Eriocheir sinensis* [24], *Macrobrachium rosenbergii* [25], *Fenneropenaeus merguiensis* [26], and *Penaeus monodon* [27]. In silico target prediction of the identified miRNAs implied that miRNAs may be involved in diverse aspects of crustacean gonadal development, such as vitellogenesis, endocrine regulation, and meiosis. Recent functional analyses of ovarian miRNAs confirmed their important roles in the ovarian development of crustaceans. In the mud crab *Scylla paramamosain*, *miR-277* has been observed to regulate the vitellogenesis-inhibiting hormone (VIH) within the eyestalks [28] and is implicated in the regulation of genes in the ERK pathway [29]. Additionally, our previous research on *P. trituberculatus* revealed that *miR-9* can influence vitellogenesis by modulating the expression of *foxl2* [30]. To date, the roles of miRNAs in the regulation of MF level are still unknown in crustaceans.

The swimming crab *P. trituberculatus* (Crustacea: Decapoda: Portunidae) is an economically significant marine fishery species prevalent in the coastal regions of Korea, Japan, China, and Southeast Asia. Moreover, it constitutes an essential part of China’s aquaculture, boasting an annual yield exceeding 100,000 tonnes [31]. Nonetheless, the rapid expansion of aquaculture, coupled with a deficit in reproduction control techniques, has led to a scarcity of high-quality seed stock, hindering industry growth. Consequently, deciphering the regulatory mechanisms of ovarian development in this species is of paramount importance. Our preceding research indicated that some miRNAs with ovary-biased expression are predicted to target *CXE2*, which encodes an enzyme responsible for MF degradation [23]. The results implied that miRNAs may play a role in regulating ovarian development by controlling the degradation of MF, the key hormone in vitellogenesis in *P. trituberculatus*. To test the hypothesis, we identified the miRNAs potentially regulating the enzymes responsible for MF degradation and established the regulatory relationship with in vitro and in vivo experiments. The results will further the understanding of miRNA-mediated mechanisms for ovarian development in crustaceans and provide valuable insights for devising novel reproduction management strategies for *P. trituberculatus*.

## 2. Results

### 2.1. In Silico Prediction of the miRNAs Targeting CXEs

**Figure 1:**
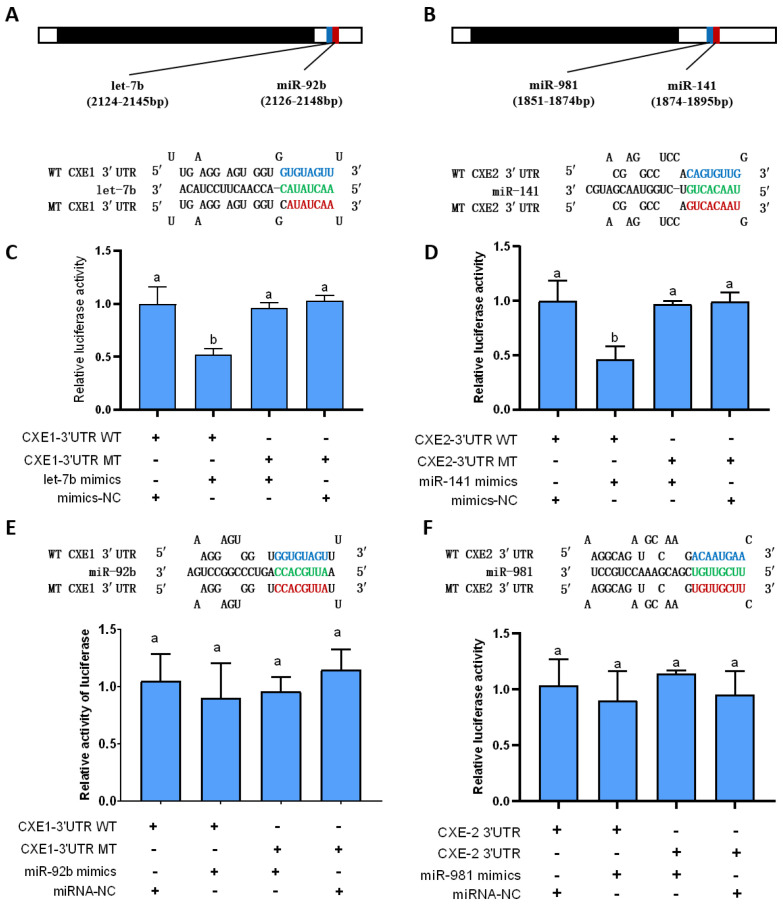
Assessment of miRNA-target interactions via dual luciferase reporter assay. Panel (**A**) shows the binding sites of *let-7b* and *miR-92b* in *CXE1* 3′ UTR. Panel (**B**) shows the binding sites of *miR-981* and *miR-141* in *CXE2* 3′ UTR. Panel (**C**) presents the dual luciferase reporter assay results for the interaction between *let-7b* and *CXE1*. Panel (**E**) shows the assay for *miR-92b* and *CXE1* interactions. Panel (**D**) illustrates the assay findings for *miR-141* and *CXE2*, while Panel (**F**) displays the results for *miR-92b* and *CXE2*. Statistical significance was determined using one-way ANOVA with subsequent Tukey’s multiple comparison tests. Distinct letters above values denote significant differences (*p* < 0.05). In these assays, HEK293T cells were co-transfected with either miRNA mimics or control mimics, along with pmirGLO vectors that contain either the wild-type (WT) (blue letters) or mutant (MT) (red letters) miRNA binding sites. Seed sequences were indicated in green. The data are shown as mean ± SD, *n* = 3.

To predict the miRNAs targeting *CXEs*, the predominant MF degradative enzymes, we used two algorithms, RNAhybrid [32] and miRanda [33]. In total, four miRNAs were predicted to bind to the 3′ UTR of *CXE1* and *CXE2*. Among them, three miRNAs were identified by both of these two algorithms, including *let-7b* vs. *CXE1*: RNAhybrid mfe: −23.1 kcal/mol, miRanda mfe: −20.2 kcal/mol; *miR-141* vs. *CXE2*: RNAhybrid mfe: −22.4 kcal/mol, miRanda mfe: 20.8 kcal/mol; *miR-981* vs. *CXE2*: RNAhybrid mfe: −24.1 kcal/mol, and miRanda mfe: −18.6 kcal/mol, and *miR-92b* was identified by RNAhybrid (*miR-92b* vs. *CXE1*: mfe: −22.2 kcal/mol) (Figure 1A,B).

### 2.2. In Vitro Validation of the Interaction between the miRNAs and Their Target Genes

To verify the binding of the predicted miRNAs to *CXEs* in vitro, the dual luciferase reporter assay was performed by co-transfection of miRNA mimics and recombinant pmirGLO vectors with the wild and mutant 3′ UTR containing the predicted miRNA binding sites into HEK293T cells. When *let-7b* mimics were co-transfected with the vector containing wild 3′ UTR of *CXE1*, the relative luciferase activity decreased compared to the negative control (Figure 1C). A similar trend was observed with *miR-141* mimics and the wild-type 3′ UTR of *CXE2*, where luciferase activity was notably diminished when compared with both the negative control and mutant sequence transfections (Figure 1D). In contrast, no significant activity reduction was detected with other miRNA mimic-vector combinations targeting the wild-type 3′ UTRs of the respective genes (Figure 1E,F). These findings provide compelling evidence that *let-7b* and *miR-141* specifically bind to the 3′ UTRs of *CXE1* and *CXE2*, respectively, substantiating their direct regulatory role.

### 2.3. Expression Profile Analyses of the miRNAs and CXEs, and Variation of Hemolymph MF during Ovarian Development

**Figure 2:**
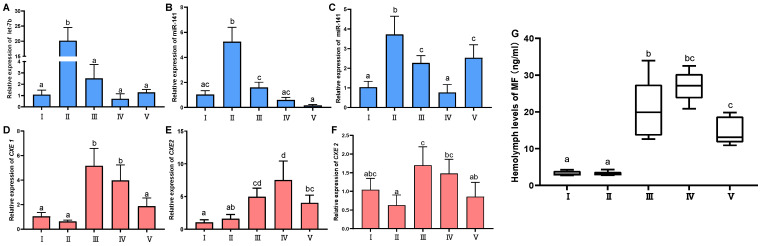
Temporal expression profiles of miRNAs (*let-7b* and *miR-141*), their target genes, and hemolymph MF levels during ovarian maturation. Panels (**A**–**C**) chart the expression patterns of *let-7b* in the hepatopancreas (**A**), *miR-141* in the hepatopancreas (**B**), and *miR-141* in the ovary (**C**). Panels (**D**–**F**) detail the relative expression of *CXE1* in the hepatopancreas (**D**), *CXE2* in the hepatopancreas (**E**), and *CXE2* in the ovary (**F**). Panel (**G**) quantifies the hemolymph MF concentrations across various stages of ovarian development (**G**). Statistical significance was determined using one-way ANOVA with subsequent Tukey’s multiple comparison tests. Distinct letters above values denote significant differences (*p* < 0.05). Data are presented as mean ± standard deviation (SD), *n* = 5.

To reveal the correlation between the expression patterns of the miRNAs (*let-7b* and *miR-141*) and corresponding their target genes (*CXE1* and *CXE2*), quantitative real-time PCR (qRT-PCR) was conducted using miRNAs isolated from hepatopancreas and ovary tissues of the female crabs at five different ovarian stages, including the previtellogenic stage (stage I), endogenous vitellogenic stage (stage II), exogenous vitellogenic stage (stage III), near-mature stage (stage IV), and mature stage (stage V). (Figure 2A–F). The results indicated significant fluctuations in the expression levels of both miRNAs and their target genes across different stages (*p* < 0.05). Specifically, *let-7b* expression surged during stage II, then significantly declined during stage III, and remained low subsequently. Conversely, *CXE1* expression was minimal at stage II and peaked at stage III. Spearman correlation analysis showed that the expression of *let-7b* and *CXE1* was negatively correlated (*r* = −0.40; *p* < 0.05). The expression of *miR-141* in both hepatopancreas and ovary tissues showed the highest expression at II and then exhibited a downward trend at later developmental stages, which was similar to the expression pattern of *let-7b*. As for *miR-141*, its expression in the hepatopancreas increased gradually during ovarian development and reached the highest level at stage IV, and its level in the ovary was the lowest at stage II and the highest at stage IV. Correlation analysis showed that there was a significant correlation in the expression between *miR-141* and *CXE2* in both hepatopancreas and ovary tissues (hepatopancreas, *r* = −0.40, *p* < 0.05; ovary, *r* = −0.43; *p* < 0.05).

The levels of hemolymph MF changed significantly at different vitellogenic stages (*p* < 0.05) (Figure 2G). The MF titer was low at stage I and stage II, then increased obviously at stage III, and remained at a high level at stage IV and stage V.

### 2.4. Effects of the miRNAs Overexpression on Target Gene Expression, MF Level, and Responsive Genes

**Figure 3:**
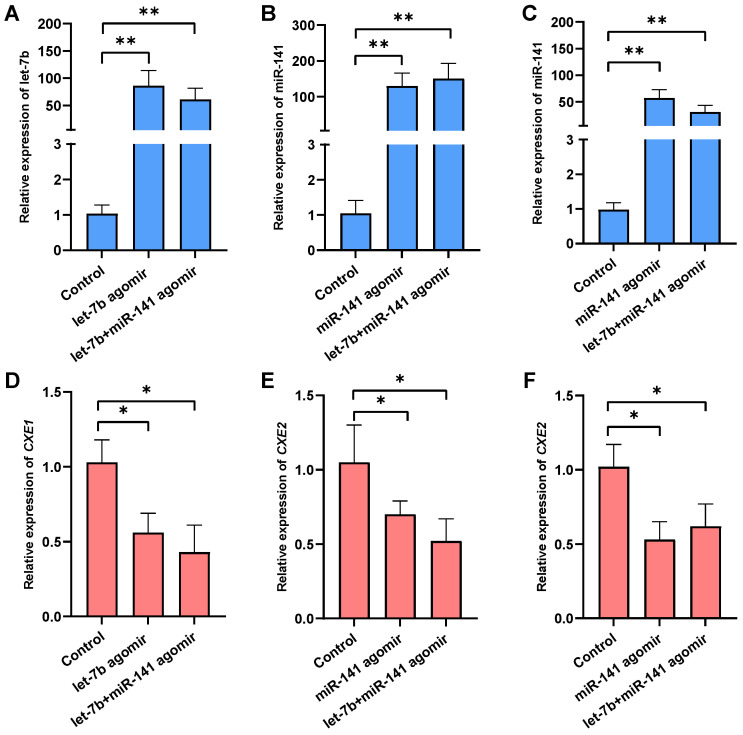
Impact of overexpressing *let-7b* and *miR-141* on target gene expression. Panels (**A**–**C**) depict the expression levels of *let-7b* in the hepatopancreas (**A**), *miR-141* in the hepatopancreas (**B**), and *miR-141* in the ovary (**C**) subsequent to agomir injection. Panels (**D**–**F**) illustrate the expression levels of *CXE1* in the hepatopancreas (**D**), *CXE2* in the hepatopancreas (**E**), and *CXE2* in the ovary (**F**) after agomir administration. Data are expressed as the mean ± SD. The means were statistically analyzed using the Student’s *t*-test, with *, *p* < 0.05 and ** *p* < 0.01. *n* = 5.

To determine the interaction between the miRNAs (*let-7b* and *miR-141*) and *CXEs* in vivo, miRNA agomir was injected into female crabs to mimic the overexpression of the miRNAs (Figure 3). The administration of *let-7b* and *miR-141* agomirs independently resulted in a substantial upregulation of *let-7b*, with an 85.16-fold increase in the hepatopancreas (*p* < 0.05), and *miR-141*, which exhibited a 128.55-fold increase in the hepatopancreas (*p* < 0.05) and a 57.16-fold increase in the ovary (*p* < 0.05). Correspondingly, the expression of *CXE1* was reduced by 44.15% in the hepatopancreas, and *CXE2* levels decreased by 30.26% in the hepatopancreas (*p* < 0.05) and 47.38% in the ovary (*p* < 0.05). Upon co-injection of *let-7b* and *miR-141* agomirs, there was an upsurge in *let-7b* expression by 60.23-fold and *miR-141* expression by 149.67-fold in the hepatopancreas (*p* < 0.05), with a 30.23-fold increase in *miR-141* in the ovary. Concurrently, *CXE1* expression in the hepatopancreas was attenuated by 57.61%, and *CXE2* expression declined by 47.28% and 38.06% in the hepatopancreas and ovary, respectively. These results corroborate the regulatory role of *let-7b* and *miR-141* on *CXE1* and *CXE2* expression within the swimming crab.

**Figure 4:**
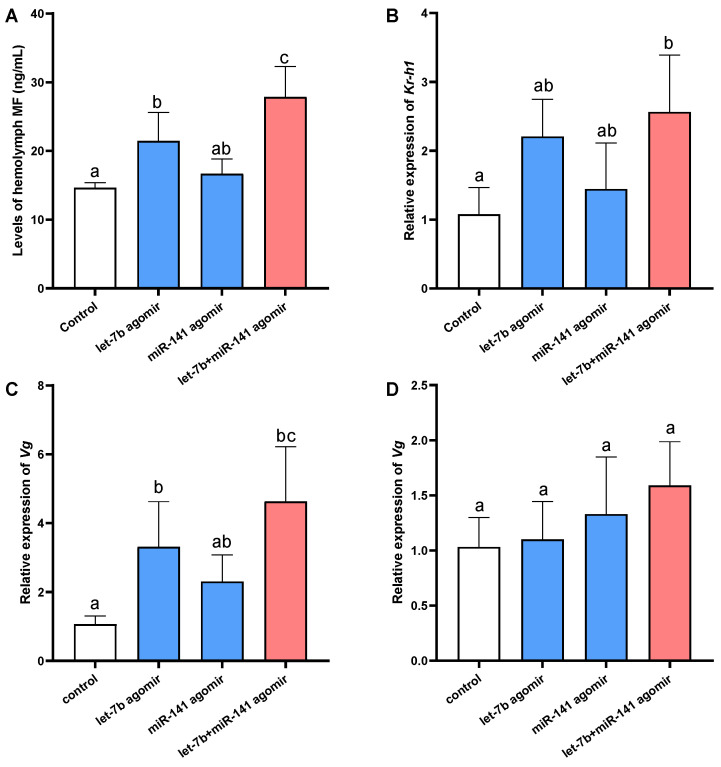
Variations in hemolymph MF levels and gene expression post *let-7b* and *miR-141* overexpression. (**A**) MF concentration in the hemolymph following agomir administration. (**B**) Quantified *Kr-h1* levels. (**C**,**D**) *Vitellogenin* expression profiles in the hepatopancreas (**C**) and ovary (**D**). Data are expressed as mean ± SD. Statistical differences were determined via one-way ANOVA and subsequent Tukey’s multiple comparison test. Distinct letters above values denote significant differences (*p* < 0.05). *n* = 5.

To further elucidate the functions of the miRNAs in vitellogenesis, we analyzed the hemolymph MF level and expression of *vitellogenin* and *Kr-h1*, an important gene responsive to MF signaling [34] (Figure 4C). Injection of *let-7b* agomir led to a significant increase in MF levels in the hemolymph, accompanied by elevated expression of *vitellogenin* in the hepatopancreas. Administration of *miR-141* agomir alone resulted in a slight, non-significant rise in MF levels, with no notable change in *Kr-h1* and *vitellogenin* expression in either the hepatopancreas or ovary. However, co-injection with *let-7b* and *miR-141* agomirs induced a marked increase in MF levels, significantly surpassing both the control and the *let-7b* agomir-only groups. In tandem with MF levels, *Kr-h1* and *vitellogenin* expressions were significantly higher in the co-injection group compared to the control. By contrast, the expression of *Kr-h1* and *vitellogenin* in ovarian tissues did not exhibit significant changes. These observations suggest that *let-7b* and *miR-141* have a synergistic effect on the regulation of MF degradation and consequently influence MF-mediated vitellogenesis.

### 2.5. Effects of MF Injection on the Expression of the miRNAs and Their Targets

**Figure 5:**
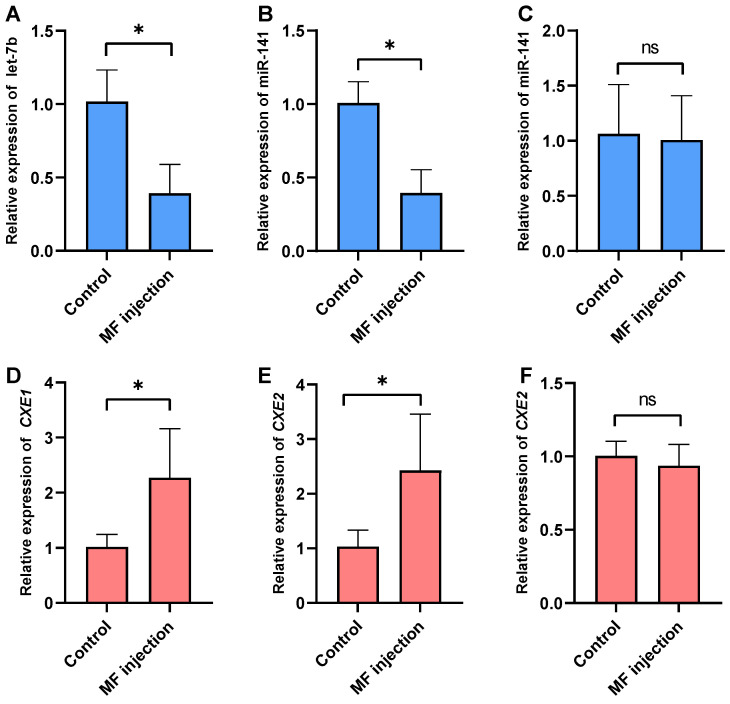
Expression levels of miRNAs and their target genes after MF injection. (**A**) Expression profile of *let-7b* in the hepatopancreas. (**B**) *miR-141* levels in the hepatopancreas. (**C**) *miR-141* expression in the ovary. (**D**) *CXE1* abundance in the hepatopancreas. (**E**) *CXE2* levels in the hepatopancreas, and (**F**) *CXE2* expression in the ovary following MF administration. The data are presented as the mean ± SD. Statistical analysis was performed using the Student’s *t*-test, with significance denoted by * *p* < 0.05, and ns indicates that there was not significant difference *n* = 3.

After MF administration, significant alterations were observed in the expression levels of *let-7b*, *miR-141*, *CXE1*, and *CXE2* within the hepatopancreas tissue (*p* < 0.05) (Figure 5). Notably, no significant changes in the expression of *miR-141* and *CXE2* were detected in the ovary. Subsequent to the injection, a marked reduction in the abundance of *let-7b* and *miR-141* in the hepatopancreas was noted (*p* < 0.05). Conversely, the expression levels of their respective target genes, *CXE1* and *CXE2*, were found to be elevated (*p* < 0.05).

## 3. Discussion

The process of vitellogenesis is a pivotal event in the development of oocytes in oviparous animals. In arthropods, miRNAs, the key post-transcriptional regulators that exert biological functions through inducing degradation or translational repression of their target mRNAs, have been demonstrated to play critical roles in regulating vitellogenin production and accumulation [35]. Research on insects has revealed that miRNAs can affect vitellogenesis by directly regulating the expression of *vitellogenin* and its receptor, and by regulating important genes in reproductive signaling pathways, such as *notch*, *kr-h1*, and *foxo* [17,18,36,37]. However, there have been only limited reports regarding the functions of miRNAs in the vitellogenesis of crustaceans. Sheng et al. reported that *miR-34* is involved in the regulation of both *vitellogenin* and *vitellogenin receptors* in *S. paramamosain* [38]. In our previous research, we found that *miR-9* regulates *vitellogenin* expression by targeting *foxl2* in *P. trituberculatus* [30]. The current study extends these findings by showing that *let-7b* and *miR-141* exhibit dynamic expression patterns during different vitellogenic stages in *P. trituberculatus*. Intriguingly, overexpression of *let-7b*, as well as a combined overexpression of *let-7b* and *miR-141*, results in a significant increase in *vitellogenin* expression in the hepatopancreas but does not significantly alter *vitellogenin* levels in the ovary. These findings underscore the regulatory impact of these miRNAs on exogenous vitellogenesis in the swimming crab, highlighting their potential role in reproductive biology.

Acting as an equivalent of JH III, MF orchestrates several physiological processes that are instrumental in ensuring reproductive success in crustaceans, including vitellogenesis [39]. It has been well documented that miRNAs are involved in the regulation of JH III levels in insects. For instance, the miRNA bantam regulates the expression of *juvenile hormone acid methyltransferase gene* (*JHAMT*), which encodes a rate-determining enzyme in JH III biosynthesis and overexpression of bantam represses *JHAMT* expression as well as JH III titer in the fly in *Drosophila melanogaster* [40]. Additionally, *let-7* is required for the coordination of the biosynthesis of JH III; the biosynthesis of JH III was influenced by the disruption and overexpression of *let-7* in the silkworm *B. mori* [41]. In *B. mori*, *miR-2766* was also predicted to regulate *juvenile hormone esterase*, which is responsible for JH III degradation [42]. These findings indicate that miRNAs can regulate JH III levels through both biosynthetic and degradative pathways in insects. In crustaceans, the regulation of MF levels is believed to be controlled by both catabolic and anabolic pathways. Recent research indicates that *miR-34* modulates FAMeT, the rate-limiting enzyme for MF biosynthesis, in the mud crab *Scylla paramamosain*. Our current study focused on the miRNA’s role in MF degradation. [43]. Using dual luciferase reporter assay, we found that *let-7b* and *miR-141* target the 3′ UTR of *CXE1* and *CXE2*, which are the genes encoding degradative enzymes of MF. Forced expression of *let-7b* and *miR-141* with agomir significantly reduces the expression of *CXE1* and *CXE2*, leading to elevated levels of hemolymph MF. Based on these in vitro and in vivo results, we demonstrate that *let-7b* and *miR-141* play a significant role in modulating MF levels in *P. trituberculatus* by targeting the degradative enzymes for MF. To the best of our knowledge, this is the first to report that miRNA can regulate MF levels by its degradative pathway.

In insects, it has been established that multiple miRNAs often work in concert to regulate the same target gene, thereby modulating vitellogenesis. In *B. mori*, *miR-2739* and *novel-miR-167* collaboratively adjust *vitellogenin receptor* expression to optimize it for ovarian development [19]. Likewise, in *L. migratoria*, clustered *miR-2*, *miR-13a*, *miR-13b,* and *miR-71* repress notch, critically contributing to JH-dependent vitellogenesis and oogenesis [18]. Another study in *L. migratoria* revealed that *let-7* and *miR-278* regulate JH-early responsive gene Kr-h1, participating in JH-stimulated vitellogenesis and egg production [17]. After co-injection of *let-7b* and *miR-141* agomirs, we observed a significantly higher hemolymph methyl farnesoate (MF) level compared to injections of either agomir *let-7b* or agomir *miR-141* alone. Correspondingly, *vitellogenin* expression was notably higher in the co-injection group than in groups receiving single miRNA agomirs. These results indicate that *let-7b* and *miR-141* synergistically modulate the orthologous genes in the same pathway (MF degradation), which in turn regulates the MF-stimulated vitellogenesis in the swimming crab. This hypothesis is further supported by the miRNA expression profile during ovarian development. Both miRNAs show a marked decrease in the hepatopancreas and ovary tissues, which are the major tissues for MF degradation at the exogenous vitellogenic stage. This reduction in miRNA expression may facilitate elevated levels of *CXEs* expression, allowing for precise control of MF levels and consequently vitellogenesis.

Interestingly, the expression of *let-7b* and *miR-141* in the hepatopancreas was down-regulated after exogenous MF injection, which was opposite to *CXEs* expression. These results suggest that a high level of MF represses *let-7b* and *miR-141* expression, and induces *CXEs* expression, to maintain the proper level of MF, which is essential for ovarian development in *P. trituberculatus*. Similar regulatory loops have also been reported for miRNAs and hormones such as JH and 20-hydroxyecdysone (20E) in insects. In *L. migratoria*, *let-7* and *miR-278* regulate the JH-responsive gene *Kr-h1*, and their expression was repressed by JH, constituting a regulatory loop of JH signaling [17]. In *D. melanogaster*, *miR-14* binds to EcR, while 20E represses *miR-14* expression, which results in enhanced EcR levels and amplified 20E signaling [44]. Similarly, in *B. mori*, *miR-281* is a regulator of *EcR-B*, and its expression is suppressed by 20E [45]. Those regulatory loops may represent the adaption of hormone-modulated gene regulation during the evolution of arthropod reproduction.

In conclusion, our work revealed the significant roles of *let-7b* and *miR-141* in the regulation of MF levels and vitellogenesis in *P. trituberculatus*. The results of both in vivo and in vitro experiments clearly showed that *let-7b* and *miR-141* can directly regulate the expression of *CXE1* and *CXE2*, the genes encoding MF degradative enzymes, at the post-transcriptional level. Using an agomir-based overexpression assay, we confirmed that these two miRNAs can cooperatively modulate MF levels by targeting *CXE1* and *CXE2.* Furthermore, we found that *let-7b* and *miR-141* are repressed by MF, which may constitute a regulatory loop of MF regulation. The findings of this study provide the first functional evidence that miRNAs can regulate the level of the important reproductive hormone, MF, by controlling its degradation. These findings extend the understanding of miRNA-mediated female reproduction regulation in crustaceans and could be pivotal for aquaculture practices, offering novel avenues for reproduction manipulation of *P. trituberculatus*.

## 4. Materials and Methods

### 4.1. Animals

The female *P. trituberculatus* used in this research were obtained from Haifeng Company, Weifang, China. To obtain crabs at different ovarian development stages, five individuals at 3 to 10 months of age were collected monthly and transferred to the laboratory where they were reared at the temperature of 23–26 °C and a salinity of 31–32 for 10 days. Before sacrifice, the crabs were placed in an ice bath until anesthetized. Then, one portion of the ovary was dissected, snap-frozen in liquid nitrogen, and stored at −80 °C. The rest of the portion was fixed in Bouin’s solution for histological examination. Based on the external features (size, morphology, and color) and histological configuration, ovarian development stages were classified into five stages. According to the stage classification, three replicates were selected for each stage.

### 4.2. Bioinformatic Prediction of the miRNAs Targeting CXEs

Two genes encoding CXEs, which are important MF degradative enzymes, have been identified in *P. trituberculatus*, namely, *CXE1* and *CXE2* [46]. To predict the miRNAs putatively targeting *CXEs*, we used RNAhybrid and miRanda programs with the miRNAs we identified in the swimming crab, which showed differential expression either between the ovary and testis or at different ovarian developmental stages.

### 4.3. Dual Luciferase Reporter Assay

In vitro target validation was performed with dual luciferase reporter assay. cDNA fragments with wild-type and mutant miRNA binding sites were artificially synthesized by General Biol Co., Ltd. (Hefei, China), cloned into pmirGLO dual luciferase reporter vector (Promega, Madison, WI, USA), and confirmed by sequencing. The constructed vectors, miRNA mimics, or the negative control were then transfected into HEK293T cells using Exfect 2000 Transfection Reagent (Vazyme Biotech Co., Ltd., Nanjing, China). After 48 h, firefly and Renilla luciferase activities were determined using the dual luciferase reporter assay system (Promega, USA). The firefly luciferase signal was normalized to the Renilla luciferase signal. All experiments were performed in three replicates.

### 4.4. miRNA Agomir

To study the function of the miRNAs in vivo, chemically modified agomirs were used to mimic the overexpression of the miRNAs. For the treatment group, each crab was injected with 200 μL of agomir at the concentration of 200 μM. For the control group, each individual was injected with an equal amount of scrambled agomir. All the injections were administered at the base of the coxa of the third walking leg. At 96 h post-injection, the crabs were anesthetized on ice and sacrificed, the hepatopancreas and ovary were collected and immediately frozen in liquid nitrogen, and the hemolymph was extracted from the sinuses at the base of the third walking leg with a syringe and used for quantification of hemolymph MF.

### 4.5. Hemolymph MF Quantification

Preparation of the hemolymph samples and detection of MF by gas chromatography-mass spectrometry (GC-MS) were performed as described by Xie et al. [6], with minor modifications. Briefly, 1 mL hemolymph sample was added into a tube containing 1.0 mL of acetonitrile and 1.0 mL of 0.9% NaCl, and then 100 ng of methyl nonadecylate (CNW, Frankfurt, Germany) was added as an internal standard. After 5 min of vortex mixing and 10 min of ultrasonic cleaning, 1 mL of hexane was added to the mixture, followed by centrifugation at 1700× *g* for 10 min. The upper phase was collected, and the remaining mixture was extracted again with 1 mL of hexane, after which the upper phases from both extractions were combined and evaporated under reduced pressure. The dried residue was dissolved in 0.1 mL of hexane. All the samples were analyzed by GC-MS with Agilent 7890A-7000B (Agilent, Santa Clara, CA, USA). GC conditions were as follows: a 30 m × 0.25 mm × μM DB-1; a temperature range of 150 °C (1 min hold) to 250 °C at a rate of 20 °C per min; and subsequent holding for 10 min. Mass-to-charge ratios (*m*/*z*) of 69, 114, 121 and 74, 87, 312 were selected to detect the MF and methyl nonadecylate, respectively. The concentration of MF in each sample was calculated according to an MF standard curve, which was drawn from a series of standard solutions (1 mL) comprising a constant weight of IS (100 ng) admixed with standard MF (Echelon, Salt Lake City, UT, USA), whose weight was 5, 20, 50, 100, and 200 ng.

### 4.6. MF Treatment

To analyze the response of the miRNAs and their targets to MF administration, the female crabs were injected with exogenous MF (Echelon, Salt Lake City, UT, USA). The individuals in the treatment group were injected with 100 μL at the concentration of 60 ng/μL, while the crabs in the control group were injected with 100 μL crustacean saline. The injections were given at the base of the coxa of the third walking leg. After 96 h, the crabs were anesthetized and dissected to collect hepatopancreas and ovary tissues for subsequent analysis.

### 4.7. RNA Extraction and qRT-PCR

Total RNA from selected tissues was extracted using TRIzol Reagent (Invitrogen, Carlsbad, CA, USA) according to the manufacturer’s protocol. Briefly, ~60 mg hepatopancreas or ovary samples were homogenized in TRIzol Reagent. The homogenates were incubated at room temperature for 5 min, and then chloroform was added, followed by vigorous shaking and a centrifugation step at 12,000× *g* for 15 min at 4 °C. The aqueous phase was carefully transferred to a new tube, and RNA was precipitated with isopropyl alcohol. After a 10 min incubation at room temperature, the samples were centrifuged at 12,000× *g* for 10 min at 4 °C. The RNA pellet was washed once with 75% ethanol, followed by centrifugation at 7500× *g* for 5 min at 4 °C. The RNA pellet was air-dried for 5 min and redissolved in RNase-free water. The quality and concentration of RNA were assessed using a spectrophotometer, and the integrity was verified through gel electrophoresis. For mRNA, cDNA was used to reverse transcribe with the FastKing RT Super Mix (Tiangen, Tianjin, China). qRT-PCR was performed with SuperReal PreMix Plus Kit (Tiangen, China) on Applied Biosystems 7500 Fast System (Applied Biosystems, Foster City, CA, USA) following the manufacturer’s instructions. The reaction system was 20 μL, containing 10.0 μL 2 × SuperReal PreMix Plus, 0.4 μL ROX Reference Dye, 0.6 μL forward and reverse primers (10 μM), 7.4 μL RNA-free water, and 1.0 μL cDNA template. The program for qPCR was as follows: 95 °C for 15 min at the initial denaturation step, 40 cycles at 95 °C for 10 s, and 60 °C for 30 s. For miRNA, cDNA was synthesized using miRcute miRNA first-strand cDNA Synthesis Kit (Tiangen, China), and qRT-PCR was performed with miRcute Plus miRNA qRT-PCR Kit (Tiangen, China). The reaction system was 20 μL, containing 10.0 μL 2 × miRcute plus miRNA Premix (SYBR&ROX), 0.4 μL forward and reverse primers (10 μM), 8.2 μL RNA-free water, and 1.0 μL miRNA first-strand cDNA. The program for miRNA qRT-PCR was 95 °C for 15 min at the initial action step, 40 cycles at 94 °C for 20 s, and 60 °C for 34 s. The relative expression levels were calculated using the 2^−ΔΔCt^ method with β-actin and U6 as the internal controls of mRNA and miRNA, respectively. Primers used for qRT-PCR are listed in Table 1.

### 4.8. Statistical Analysis

All data are shown as the mean ± SD. Statistical analyses were performed using Student’s t-test or one-way analysis of variance (one-way ANOVA) after checking for normality and homogeneity of variance in the data. If significant differences were found, Tukey’s post-hoc test was used to identify the differences. The correlation between the expression of all the tested miRNAs and the genes was performed using Spearman correlation analysis. The statistically significant level was set as *p* < 0.05.

## Figures and Tables

**Table 1:** The primers used for qRT-PCR.

Primer	Sequence (5′-3′)
β-actin-F	CGAAACCTTCAACACTCCCG
β-actin-R	GGGACAGTGTGTGAAACGCC
CXE1-F [46]	CACGGGAAAGGTGTCTGGTAT
CXE1-R [46]	TTGGCGTAGGGAATGGAGTAG
CXE2-F [46]	GCACCTTCCGCTATGAGTTT
CXE2-R [46]	CCGCCAGTGAATAGATAGAACA
Kr-h1-F [34]	TACATCCCACATCCCCAACA
Kr-h1-R [34]	CGTCACATCAGGGACCATTT
Vitellogenin-F [34]	ATTGCTGGTCGCCTGTTGT
Vitellogenin-R [34]	ATCCTGCCTCAGTCCCCTTA
U6-F U6-R	CTTGCTTCGGCAGAACATATACT AACGCTTCACGATTTTGCGT
let-7b	GCAACTATACAACCTACTACCTCA
miR-141	CAACACTGTCTGGTAAAGATGC

## Data Availability

The data presented in this study are available from the author upon request.
